# Nephroprotective Effect of Zingerone against CCl_4_-Induced Renal Toxicity in Swiss Albino Mice: Molecular Mechanism

**DOI:** 10.1155/2018/2474831

**Published:** 2018-01-30

**Authors:** Mohammed M. Safhi

**Affiliations:** Department of Pharmacology & Toxicology, Pharmacy College, Jazan University, Gizan, Saudi Arabia

## Abstract

The protective effects of Zingerone against CCl_4_ induced nephrotoxicity in Swiss albino mice via modulation of metabolizing enzyme, oxidative stress, inflammatory cytokines, and apoptosis. The biochemical estimation indicated that the BUN and creatinine were significantly increased in group 2 (CCl_4_) compared to group 1 (normal) which was significantly reduced after treatment with Zingerone in group 3 when compared with group 2. The CCl_4_ treatment has significantly increased TBARS levels and reduced the antioxidant enzyme such as GSH, GPx, GR, GST, CAT, and SOD in group 2 compared to group 1, while the Zingerone treatment showed significant reduction in TBARS levels and increased the antioxidant enzymes in group 3 (CCl_4_ + Zingerone) as compared to group 2. Similarly, it was observed that CCl_4_ significantly increased the cytokines such as IL-1*β*, IL-2, and TNF*α* levels in group 2 as compared to group 1. The treatment with Zingerone significantly attenuated the levels of IL-1*β*, IL-2, and TNF*α* in group 3 compared to group 2. Caspase 3 and caspase 9 were also significantly increased in CCl_4_-treated group 2, whereas Zingerone treatment significantly reduced the elevated levels of caspases 3 and 9 in group 3 compared to group 2.

## 1. Introduction

Chemical- and drug-induced nephrotoxicity is a leading cause of acute kidney injury which may be due to altered intraglomerular hemodynamic, inflammation, rhabdomyolysis, microangiopathy, and tubular cell toxicity. It is recognized as main causes of mortality and morbidity [1]. Carbon tetrachloride (CCl_4_) is commonly known by many other names like tetracholoromethane, Halon-104, and Refrigerant-10 [2]. It is widely used in fire extinguishers, refrigerants, and cleaning agents. CCl_4_ is one of the most potent toxins, which is widely used in scientific research to produce experimental model that mimic the oxidative stress in many pathophysiological situation [2]. The toxicity produced by CCl_4_ depends on the generation of trichloromethyl radical (CCl_3_^•^), which further converts trichloromethyl into trichloromethyl peroxyl radical (CCl_3_O_2_^•^) in the presence of oxygen that is more toxic than trichloromethyl radicals [3]. These radicals cause peroxidative degeneration of many tissues by binding with lipids, proteins, and DNA. Several previous research studies have indicated that CCl_4_ is the best model for the generation of reactive oxygen species (ROS) in many tissues [4]. Oxidative stress develops due to the generation of ROS such as superoxide anion, hydroxyl radical, hydrogen peroxide, and other radicals after CCl_4_ administration [5]. Cytokines are small proteins that are involved in cell signalling such as autocrine, paracrine, and endocrine signalling. They play an important role in all aspects of inflammation and immunity including cell proliferation, maturation, migration, adhesion, and so on. Cytokines IL-1*β*, IL-2, IL-6, and TNF*α* are produced by activation of macrophages, and they are involved in the upregulation of inflammation. [6]. Apoptosis is the molecular process of cell death that occurs in organism in which programmed sequence of events leads to the elimination of cell without releasing harmful substance in surrounding area. It has an effective role in developing and maintaining the health of the body by eliminating old cells and unhealthy cells. Stimulation and inhibition of apoptosis can play a role in many diseases. Apoptosis leads to several changes in cell morphology including cell shrinkages, DNA fragmentation, and messenger RNA decay, which is highly regulated process by which cell dies. The mechanism of cell death is due to the activation of initiator caspase (caspase 9) that consequently activates executioner caspase (caspase 3) which finally kills the cell by the degradation of proteins [7].

Several research studies have been conducted on many herbal medicines to protect the CCl_4_ intoxication by improving the antioxidant enzymes such as superoxide dismutase, catalase, GPx, GR, and GST and also by enhancing the reduced glutathione content. Medicinal plants are playing a significant role against different types of diseases from the ancient time. Ginger is one the most popular plants for condiment and natural drugs for various pharmacological actions. Zingerone is the main active constituent present in ginger (*Zingiber officinale*). It is nontoxic with varied pharmacological activities like anti-inflammatory, antioxidant, anticancer, lipolytic, and antiemetic [8]. Previous studies have reported that Zingerone played important roles such as inhibiting radiation-induced decline in endogenous antioxidants, scavenging free radicals, and protecting brain mitochondria against tellurium toxicity [9, 10]. Therefore, in this study, I have selected Zingerone to elucidate its protective effect against CCl_4_-induced nephrotoxicity in mice.

## 2. Materials and Methods

### 2.1. Chemicals and Drugs

Thiobarbituric acid (TBA), CDNB, DTNB, glutathione (oxidized and reduced), NADPH, and Zingerone were procured from Sigma-Aldrich Co., USA. Interleukin (IL-1*β*-ab197742 and IL-2-ab46096 and TNF*α*-ab100747) and apoptosis kits (caspases 3-ab39401 and caspases 9-ab65608) were purchased from Abcam through Abdullah Favad Holding Company, Dammam, Kingdom of Saudi Arabia.

### 2.2. Animal Acclimatization

Male Swiss albino mice weighing 35–50 g and aged 4-5 months were taken for this study. The mice were procured from the Medical Research Centre Animal House, Jazan University, Jazan, and were kept at the College of Pharmacy animal house with light-dark cycle (12 hrs), at a temperature of 23 ± 2°C. All animals were given standard basal diet and water ad libitum. Animal care handling was practiced according to the Institutional Animal Care and Use Committee (IACUC) guidelines for the care and use of laboratory animals [11, 12].

### 2.3. Experimental Scheme

The mice were randomly divided into four groups each having eight animals. Group 1 was normal control and received normal saline only. Group 2 was toxic, given dose of CCl_4_ (1 : 1 *v/v*) solution in olive oil/paraffin oil containing 1.5 ml/kg intraperitoneal injections twice a week for 15 days. Group 3 received pretreatment with Zingerone (100 mg/kg) orally for 15 days and, on the last day, was given CCl_4_. Group 4 was only given 100 mg/kg Zingerone orally for 15 days. On the 16th day, blood samples were collected and serum was isolated from blood and the marker of kidney function test (creatinine and blood urea nitrogen) was performed using Crescent Diagnostic Kits, KSA. Further, all the mice were sacrificed under light ether anaesthesia and the kidney was isolated from each animal and stored in freezer for further analysis. The kidney was weighed and homogenized in 10 mM Tris-HCl, pH 7.4 to give a 10% (*w/v*) homogenate, and centrifuged at 3000 rpm for 5 min at 4°C to isolate the supernatant (S1) for the assay of lipid peroxidation (LPO). Further remaining homogenates were also centrifuged at 12500*g* for 30 min to get the post mitochondrial supernatant (PMS) for further antioxidant enzyme analysis.

### 2.4. Oxidative Stress Analysis

The thiobarbituric acid reactive substance (TBARS) estimation was conducted using the method of Utley et al. [13] modified by Safhi et al. [14], and the absorbance was monitored at 535 nm. Glutathione (GSH) was measured using the method of Jollow et al. [15] with slight modification, and the color absorbance was monitored at 412 nm. The glutathione peroxidase (GPx) estimation was completed as per the method of Mohandas et al. [16]. Glutathione reductase (GR) was estimated as per the method of Carlberg and Mannervik [17] which is slightly modified by Islam et al. [18]. The activity of catalase (CAT) was measured according to the method of Claiborne [19], and changes in absorbance were recorded at 240 nm. Superoxide dismutase (SOD) estimation was carried out as per Stevens et al. [20], and absorbance was recorded at 480 nm. Protein estimation was done by the method of Lowry et al. [21].

### 2.5. Inflammatory Cytokines (IL-1*β*, IL-2, and TNF*α*) and Apoptosis (Caspase 3 and Caspase 9)

#### 2.5.1. Assay

The kidney tissue supernatant was assayed by using Abcam's assay kit for the measurement of IL-1*β*, IL-2, and TNF*α*. For each test, standard curve was plotted and followed the assay kit guideline.

#### 2.5.2. Interleukin 1*β*

Interleukin 1*β* in tissue sample was assessed by a simple step sandwich ELISA assay as per standard protocol given by the kits. The end reaction was quantified by the intensity of colour formation which was measured at 450 nm by using BioTek ELX800 ELISA reader. The sample concentration was calculated by extrapolating on the standard curve.

#### 2.5.3. Interleukin 2

The sandwich ELISA was used to quantify interleukin 2 in tissue samples. The assay employs a monoclonal antibody specific to IL-2 coated on a 96-well assay plate. The standard and sample were analysed by binding with immobilized antibody. The end reaction was quantified by the intensity of colour formation which was measured at 450 nm by using BioTek ELX800 ELISA reader. The sample concentration was calculated by extrapolating on the standard curve.

#### 2.5.4. TNF*α*

TNF*α* is an apoptotic marker which was determined in tissue sample by an *in vitro* sandwich ELISA assay. The assay employs a specific antibody coated on a 96-well titre plate. The standard and sample were analysed by binding with immobilized antibody. The end reaction was quantified by the intensity of colour formation which was measured at 450 nm by using BioTek ELX800 ELISA reader. The sample concentration was calculated by extrapolating on the standard curve.

#### 2.5.5. Caspase 3

Caspase 3 is an apoptotic marker which was determined in tissue sample by spectrophotometric detection using chromophore p-nitroaniline (p-NA). The end reaction was quantified by p-NA light emission which was measured at 405 nm using BioTek ELX800 ELISA reader. The sample concentration was calculated by extrapolating on the standard curve.

#### 2.5.6. Caspase 9

Caspase 9 is an apoptotic marker which was determined in tissue sample by spectrophotometric detection using chromophore p-nitroaniline (p-NA) after cleaving the labelled substrate LEHD-p-NA. The end reaction was quantified by detecting free p-NA after cleavage from the substrate, which was measured at 405 nm by using BioTek ELX800 ELISA reader. The sample concentration was calculated by extrapolating on the standard curve.

### 2.6. Statistical Analysis

The results were collected and expressed as average ± standard error of mean, and comparison between groups was carried out by one-way ANOVA. *p* < 0.05 was considered to be significantly statistical.

## 3. Results

### 3.1. Effect of CCl_4_ on Kidney Function Test (Creatinine and BUN) in Serum and Its Treatment with Zingerone

**Figure 1:**
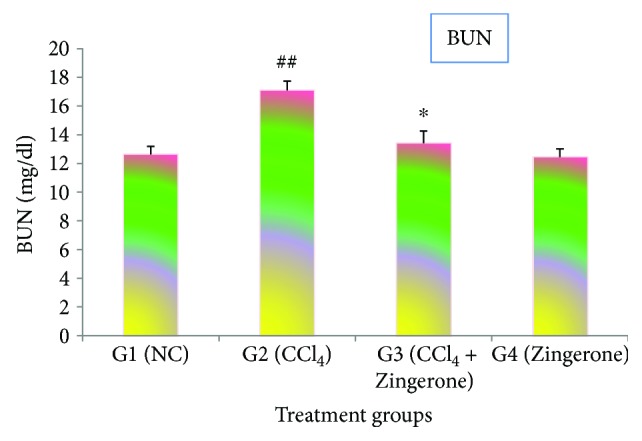
Effect of Zingerone on BUN level in the serum of CCl_4_-treated mice. ^##^*p* < 0.001 versus NC group 1 and ^∗^*p* < 0.01 versus CCl_4_-treated toxic group 2.

**Figure 2:**
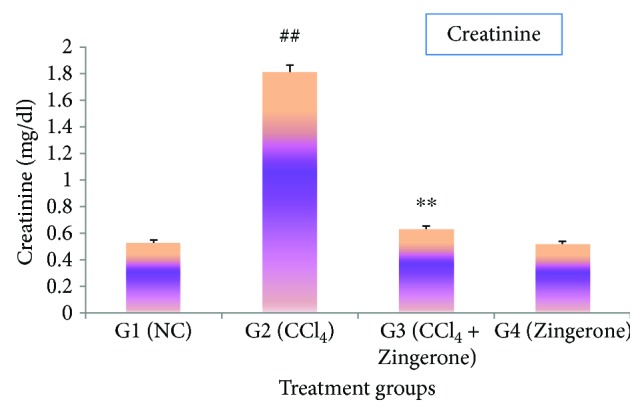
Effect of Zingerone on creatinine level in the serum of CCl_4_-treated mice. ^##^*p* < 0.001 versus NC group 1 and ^∗∗^*p* < 0.001 versus CCl_4_-treated toxic group 2.

The present investigation showed that the administration of CCl_4_ to mice produced a significant increase (*p* < 0.001) in the serum creatinine and blood urea nitrogen (BUN) levels in group 2 as compared to group 1. In contrast, the pretreatment with Zingerone at a dose of 100 mg/kg significantly depleted the creatinine and BUN levels in group 3 as compared to group 2 (Figures 1 and 2). The treatment of Zingerone alone (100 mg/kg) to group 4 was showing similar type of effects like group 1.

### 3.2. Effect of CCl_4_ on TBARS and Its Treatment with Zingerone

**Figure 3:**
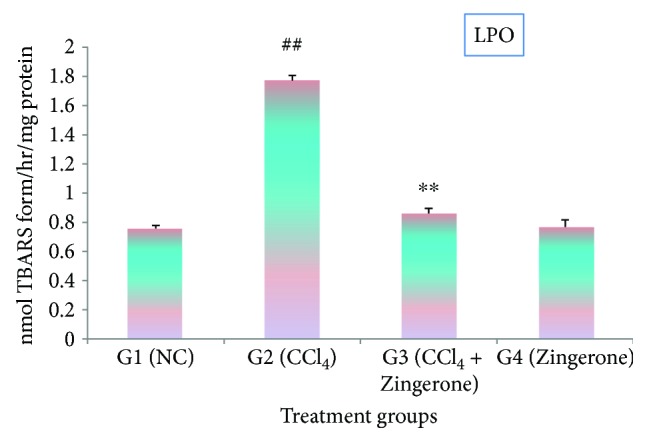
Effect of Zingerone on LPO levels in the kidney tissue of CCl_4_-treated mice. ^##^*p* < 0.001 versus NC group 1 and ^∗∗^*p* < 0.001 versus CCl_4_-treated toxic group2.

The effect of CCl_4_ on lipid peroxidation and its treatments with Zingerone is depicted in Figure 3. The levels of thiobarbituric acid reactive substances (TABRS) were elevated significantly (*p* < 0.001) in CCl_4_-treated group 2 compared to group 1. The treatment with Zingerone at a dose of 100 mg/kg showed protection by decreasing the levels of TBARS significantly in group 3 compared to group 2. There was no significant change in the TBARS level which was recorded after treatment with Zingerone (100 mg/kg) alone to group 4 as compared to group 1.

### 3.3. Effect of CCl_4_ on Glutathione (GSH) Content and Its Treatment with Zingerone

**Figure 4:**
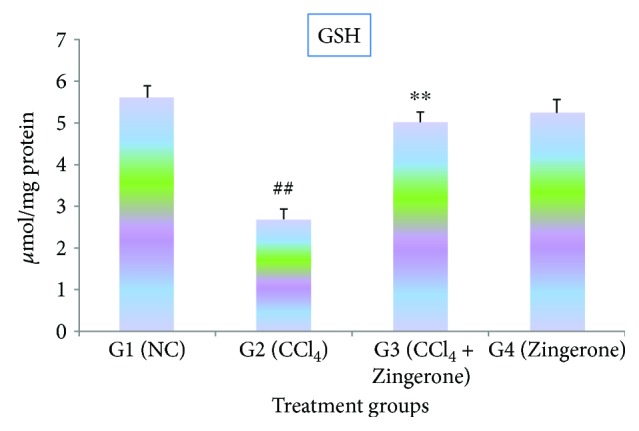
Effect of Zingerone on GSH levels in the kidney tissue of CCl_4_-treated mice. ^##^*p* < 0.001 versus NC group 1 and ^∗∗^*p* < 0.001 versus CCl_4_-treated toxic group 2.

Figure 4 representing that glutathione content was significantly (*p* < 0.001) reduced after the treatment with CCl_4_ in group 2 when compared to group 1. The administration of Zingerone (100 mg/kg) raises the content of GSH significantly in group 3 compared to group 2 and showed protection. No significant difference was recorded when only Zingerone (100 mg/kg) was given to group 4 as compared to group 1.

### 3.4. Effect of CCl_4_ on Antioxidant Enzyme and Its Treatment with Zingerone

CCl_4_ significantly (*p* < 0.001) decreased the antioxidant enzymes (GPx, GR, GST, CAT, and SOD) in group 2 when compared to group 1. The treatment with Zingerone (100 mg/kg) enhances the levels of antioxidant enzymes (GPx, GR, GST, CAT, and SOD) in group 3 compared to group 2 as shown in Table 1. The treatment with only Zingerone at a dose of 100 mg/kg to group 4 did not elicit any significant difference in the activity of abovementioned enzymatic antioxidants when compared to group 1.

### 3.5. Effect of CCl_4_ on IL-1*β*, IL-2, and TNF*α* and Its Treatment with Zingerone

**Figure 5:**
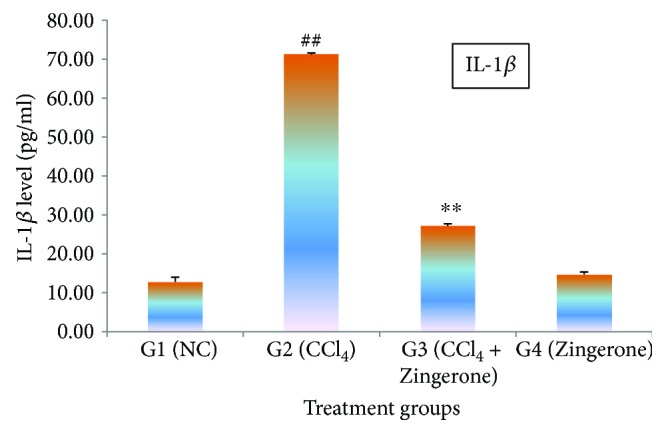
Effect of Zingerone on IL-1*β* levels in the kidney tissue of CCl_4_-treated mice. ^##^*p* < 0.001 compared with NC group 1 and ^∗∗^*p* < 0.001 compared with CCl_4_-treated toxic group 2.

**Figure 6:**
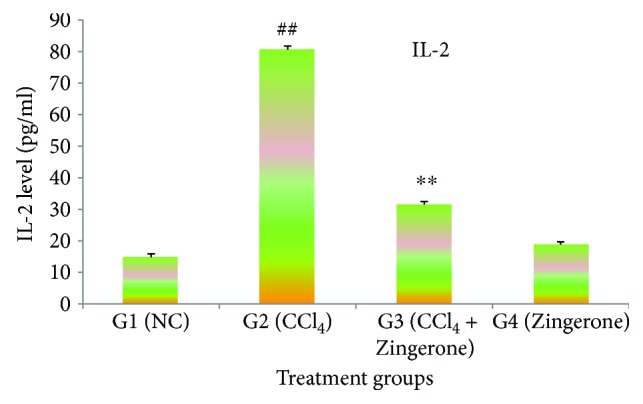
Effect of Zingerone on IL-2 levels in the kidney tissue of CCl_4_-treated mice. ^##^*p* < 0.001 compared with NC group 1 and ^∗∗^*p* < 0.001 compared with CCl_4_-treated toxic group 2.

**Figure 7:**
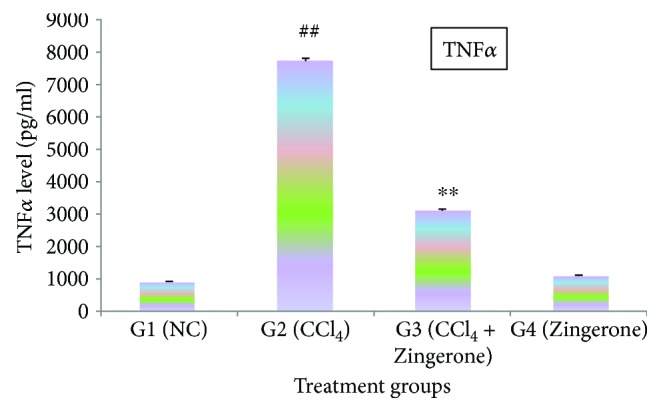
Effect of Zingerone on TNF*α* levels in the kidney tissue of CCl_4_-treated mice. ^##^*p* < 0.001 compared with NC group 1 and ^∗∗^*p* < 0.001 compared with CCl_4_-treated toxic group 2.

In this study, the cytokines IL-1*β*, IL-2, and TNF*α* were significantly (*p* < 0.001) elevated in group 2 treated with CCl_4_ when compared with group 1, while treatment with Zingerone (100 mg/kg) significantly depleted the elevated levels of IL-1*β*, IL-2, and TNF*α* in group 3 when compared to group 2. When Zingerone was administered alone in a dose of 100 mg/kg to group 4, no significant changes in the levels of abovementioned inflammatory cytokines were observed when compared with group 1 (Figures 5–7).

### 3.6. Effect of CCl_4_ on Caspase 3 and Caspase 9 and Its Treatment with Zingerone

**Figure 8:**
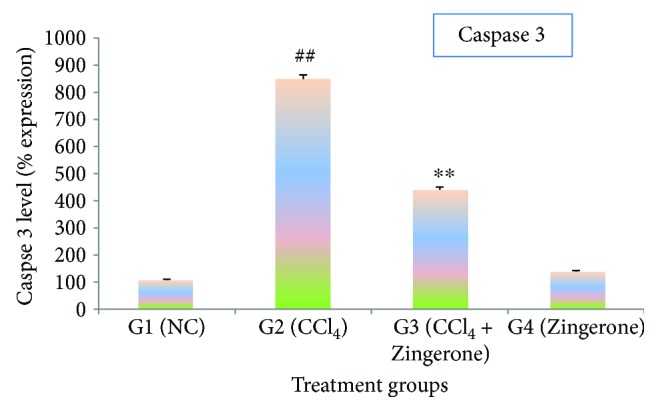
Effect of Zingerone on caspase 3 levels in the kidney tissue of CCl_4_-treated mice. ^##^*p* < 0.001 compared with NC group 1 and ^∗∗^*p* < 0.001 compared with CCl_4_-treated toxic group 2.

**Figure 9:**
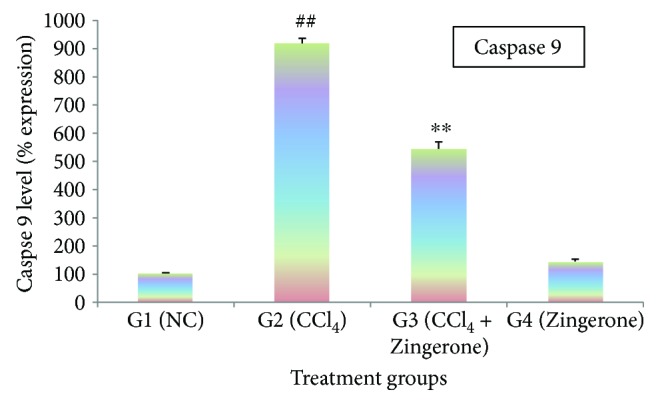
Effect of Zingerone on caspase 9 levels in the kidney tissue of CCl_4_-treated mice. ^##^*p* < 0.001 compared with NC group 1 and ^∗∗^*p* < 0.001 compared with CCl_4_-treated toxic group 2.

CCl_4_ treatment triggered a significant (*p* < 0.001) elevation of caspase 3 and caspase 9 activity in group 2 as compared to group 1, while the treatment with Zingerone (100 mg/kg) significantly reduced the activity of caspase 3 and caspase 9 in group 3 when compared to group 2. The administration of Zingerone (100 mg/kg) alone in group 4 showed no significant changes in the activity of caspases as compared to group 1 (Figures 8 and 9).

## 4. Discussion

Chemical- and drug-induced acute kidney injury is a major cause of death worldwide. Previous published research studies have reported that CCl_4_ exposure causes damage to the kidney due to enhanced production of reactive oxygen species [22, 23]. Several natural products have been demonstrated to possess antioxidant properties and are capable of suppressing the generation of free radicals to protect acute kidney damage [22, 24]. The present investigation showed that administration of CCl_4_ markedly increased the levels of creatinine and blood urea nitrogen (BUN). Creatinine and BUN are the nitrogenous end product of metabolism in the blood, distributed throughout the total body water and are normally removed from blood by the kidney. After the exposure of CCl_4_, the kidney function slows down and increases the levels of creatinine and BUN in the blood. The treatment with Zingerone significantly reduced the increased levels of creatinine and BUN.

Many research studies have demonstrated that CCl_4_ intoxication is the major source of free radical generation in many tissues such as the liver, kidney, lungs, brain, and blood [25]. It has also been reported that after CCl_4_ administration to rats, the level of CCl_4_ is distributed at higher concentration in the kidney than in the liver [26], since the kidney has high affinity for CCl_4_ and contains cytochrome P_450_ predominantly in the cortex. The most common free radicals from CCl_4_ is trichloromethyl radical (CCl_3_^•^) and trichloromethyl peroxyl radical (CCl_3_O_2_^•^) [3]. These radicals bind to intracellular protein, lipids of cell membrane, and DNA resulting in protein denaturation, lipid peroxidation, and oxidative DNA damage that leads to cell death [27, 28]. Lipid peroxidation is one of the important markers of oxidative stress. The level of TBARS was found to be significantly increased in kidney tissue after administration of CCl_4_. Zingerone treatment has shown a marked diminution in TBARS level. This may be due to antioxidant properties of Zingerone, which scavenges free radicals thereby inhibiting lipid peroxidation.

Glutathione (GSH) is an important antioxidant which shows an important role in stopping the injury to cellular components caused by free radicals and peroxides [29]. CCl_4_ indicated the marked reduction in GSH content due to impairment of H_2_O_2_ clearance and promotion of hydroxyl radical (^•^OH) formation which leads to oxidative stress [30, 31]. The effective restoration of lipid peroxidation and enhancement of glutathione content were observed after the treatment with Zingerone.

Superoxide dismutase (SOD) is metalloenzyme that catalyses the dismutation of superoxide radicals and converts it into H_2_O_2_ [32, 33]. SOD activity was significantly decreased after treatment with CCl_4_, whereas administration of Zingerone restored the decreased activity of SOD. Catalase is another antioxidant enzyme which is responsible for the reduction of H_2_O_2_ to water and oxygen thus preventing the damage of cells from the oxidative stress. CCl_4_ decreased the activity of CAT which was protected significantly with Zingerone treatment.

GPx is an important antioxidant enzyme which plays a leading role in eliminating excess free radicals and lipid hydroperoxides from the cell with the help of GSH, which in turn is oxidized to glutathione disulfide (GSSG) after donating proton. GR utilizes the NADPH and maintains the GSH in a reduced form by converting GSSG back to GSH, thus maintaining the pool of GSH. CCl_4_ treatment has significantly reduced the GSH content and modified the activity of various important enzymes such as GPx, GR, and GST. Decreased content of GSH may be responsible for the decreased activity of glutathione-metabolizing enzymes in the kidney tissue. Previous report also showed that CCl_4_ changed the activity of these enzymes which play a major role in scavenging toxic-free radicals [34]. The activity of GPx, GR, and GST was reduced after CCl_4_ treatment, while treatment with Zingerone has significantly augmented the activity of these enzymes in the kidney tissue.

Oxidative stress and inflammatory cytokines are closely linked and play an important role in chemical- and drug-induced acute kidney damage. The current finding emphasised on the detailed investigation of molecular marker for inflammatory cytokines (IL-1*β*, IL-2, and TNF*α*) and apoptosis (caspase 3 and caspase 9) in the kidney tissues. Increase in oxidative damage increases the production of inflammatory cytokines or vice versa. This two-way relationship between oxidative stress and inflammatory cytokines has been reported by several researchers [35]. CCl_4_ is responsible for the generation of toxic trichloromethyl radical (CCl_3_^•^) and trichloromethyl peroxyl radical (CCl_3_O_2_^•^) which may be accountable for the production of cytokines such as IL-1*β*, IL-2, and TNF*α*. These cytokines are released by the leukocytes and renal tubular cell and are associated with the pathogenesis of inflammation in acute kidney damage. The inflammatory processes are mainly activated by NF-*κ*B, which modulate cytokine production and therefore increased the production of inflammatory cytokines [36]. IL-1*β* is a precursor protein which is activated by caspase 1 and a key cytokine involving in the renal damage. It produces inflammation by the activation of cyclooxygenase-2 (COX-2) and releasing PGE_2._ IL-2 has dual function inflammatory and anti-inflammatory responses. IL-2 is released by Th_1_ cells that proliferate CD4+ T cells which stimulate B cell induction. However, the other side of IL-2 stimulates cytotoxic effect due to the induction of CD8+ T cells and also produces tumour necrosis factor (TNF*α*) and interferon gamma. Furthermore, IL-2 involved in the development of regulatory T cells and suppressing the effector Th_17_ cells leads to Fas-mediated apoptosis. TNF*α* is a predominant cytokine in the inflammatory tissue damage and plays an important role in nephron toxicity. It plays an immune governing function to conserve homeostasis of the immune system. The present study revealed that CCl_4_ increased the production of classic inflammatory cytokines such as IL-1*β*, IL-2, and TNF*α*. The increased levels of inflammatory cytokines might depend on the CCl_4_, or its toxic-free radicals (CCl_3_^•^ and CCl_3_O_2_^•^) induced NF-*κ*B activation. The elevated levels of inflammatory cytokines (IL-1*β*, IL-2, and TNF*α*) were reduced to near normal levels after the administration of Zingerone.

Apoptosis is a programmed cascade of enzymatic event that accomplish cell death and involved in the clearance of infiltrating inflammatory cytokines which is beneficial in renal toxicity. In opposition, in appropriate activation of apoptosis due to activation of caspases may further promote renal toxicity by damaging the tubular cells [37]. Caspases are involved in apoptosis which have been subclassified by their mechanism of action based on either initiator caspases like caspase 9 or executioner caspase 3. Initiator caspase activates executioner caspase that subsequently coordinates their activities to downstream the key structural protein Bax/Bcl-2 and plays key role in the accomplishment of apoptosis [38]. CCl_4_ treatment has increased the activity of caspase 9 and caspase 3, which may promote apoptosis by stimulating proapoptosis Bax and inhibiting antiapoptosis Bcl-2 proteins. The activity of these caspases like caspases 3 and 9 significantly diminished after Zingerone treatment.

## 5. Conclusion

The present study demonstrated that CCl_4_ is a potent nephrotoxic substance, which leads to oxidative stress by depleting the activities of antioxidant enzymes, inflammatory cytokine production, and stimulate apoptosis. The treatment with Zingerone significantly attenuated the CCl_4_-induced renal toxicity. Therefore, Zingerone can be used as an effective therapeutic agent for the treatment of drug-induced nephrotoxicity.

## Figures and Tables

**Table 1:** Effect of Zingerone on the activity of enzymatic antioxidants in the kidney tissue of Swiss albino mice treated with CCl_4_.

Treatment	nmol NADPH oxidized/min/mg protein		GST (nmol CDNB conjugates formed/min/mg protein)	CAT (nmol H_2_O_2_ consumed/min/mg protein)	SOD (nmol (−) epinephrine protected from oxidation/min/mg protein)
	GPx	GR			
G1 (NC)	46.36 ± 3.27	35.83 ± 2.14	0.66 ± 0.04	1.241 ± 0.07	1.97 ± 0.11
G2 (CCl_4_)	25.36 ± 1.56^##^	19.87 ± 1.04^##^	0.23 ± 0.02^##^	0.71 ± 0.02^##^	0.85 ± 0.05^##^
G3 (CCl_4_ + Zingerone)	41.38 ± 1.36^∗∗^	31.80 ± 1.80^∗^	0.52 ± 0.04^∗∗^	1.07 ± 0.06^∗^	1.75 ± 0.14^∗∗^
G4 (Zingerone)	43.53 ± 2.12	33.91 ± 2.29	0.61 ± 0.05	1.12 ± 0.09	1.72 ± 0.18

^##^ *p* < 0.001 versus group 1 (normal control). ^∗^*p* < 0.01 and ^∗∗^*p* < 0.001 versus group 2 (CCl_4_ treated).
